# Bilateral uveitis and Usher syndrome: a case report

**DOI:** 10.1186/s13256-015-0534-7

**Published:** 2015-03-15

**Authors:** Matthew D Benson, Ian M MacDonald

**Affiliations:** Department of Ophthalmology and Visual Sciences, University of Alberta, Room 2319, 10240 Kingsway Avenue, Edmonton, AB T5H 3V9 Canada

**Keywords:** Granulomatous uveitis, Keratic precipitates, Retinitis pigmentosa, Usher syndrome

## Abstract

**Introduction:**

Usher syndrome is a genetically heterogeneous condition and represents the most common cause of inherited combined vision and hearing loss. Deficits manifest as sensorineural hearing loss that typically develops at a young age and retinitis pigmentosa that can lead to peripheral vision loss and night blindness. As a result, this syndrome can have a significant impact on a patient’s quality of life.

Previous studies have described an association between Usher syndrome and Fuchs’ heterochromic iridocyclitis, a form of non-granulomatous uveitis that generally presents in a unilateral manner. We present a rare finding of bilateral uveitis and, to the best of our knowledge, the first report of granulomatous uveitis as a feature in a patient with Usher syndrome.

**Case presentation:**

A 45-year-old Caucasian woman with a known history of retinitis pigmentosa presented to our clinic with suspected Usher syndrome, given her report of long-standing hearing loss. Aside from a mild loss in visual acuity, our patient was otherwise asymptomatic. Visual field testing, audiology and electroretinography findings supported the diagnosis of Usher syndrome. With slit lamp examination she was found to have bilateral keratic precipitates, with large, greasy-white, mutton-fat keratic precipitates on the endothelial surface of her left eye. A thorough work-up that included blood tests and imaging was negative for an alternative cause of her uveitis.

**Conclusion:**

We present a rare finding of bilateral uveitis and what we believe to be the first reported instance of mutton-fat keratic precipitates and granulomatous uveitis as a feature in a patient with Usher syndrome. By identifying atypical presentations of the disease, we hope to contribute to the range of ophthalmic conditions that may be seen in association with Usher syndrome.

## Introduction

Usher syndrome represents a genetically diverse condition that involves both early-onset sensorineural hearing loss and retinal pathology. While reports of disease prevalence vary, the condition has been estimated to occur in three in 100, 000 individuals. Moreover, Usher syndrome is the most common cause of combined deafness and blindness, accounting for nearly 50% of all cases [1-3].

There exist three distinct types of Usher syndrome that differ based on the onset and severity of symptoms. Type 1 includes individuals with profound deafness at birth with the onset of visual disturbances occurring prior to puberty. This is the most severe form of disease and is often accompanied by vestibular dysfunction. Type 2 involves congenital hearing loss that is less severe than type 1, with the onset of retinal disease occurring in the first or second decade of life. Finally, Type 3 is the least common presentation and involves variable onset of both hearing and vision difficulties [2].

Currently, there are a number of genes with causative roles in Usher syndrome. These genes are inherited in an autosomal recessive manner and encode for peptides that form a complex in sensory hair cilia of the inner ear and in photoreceptors of the retina. Mutations in these genes lead to sensorineural hearing loss that is generally non-progressive and retinal changes consistent with retinitis pigmentosa (RP) [1].

Uveitis is a form of ocular inflammation that can involve the iris, ciliary body and choroid, and is subdivided into granulomatous and non-granulomatous types. These types may be differentiated based on the inflammatory cells present: granulomatous uveitis involves macrophages and epithelioid cells and non-granulomatous uveitis is predominantly defined by a lymphocyte and plasma cell response [4]. To date, several studies have demonstrated an association of RP with a form of non-granulomatous uveitis called Fuchs’ heterochromic iridocyclitis (FHIC) [5-9]. This form of anterior uveitis is characterized by stellate endothelial keratic precipitates (KPs) and atrophic changes in the iris. More recently, two studies have confirmed an association between Usher syndrome and FHIC [10,11]. Furthermore, a recent study reported a case of non-granulomatous uveitis that was distinct from FHIC in a patient with Usher syndrome [3].

While associations between Usher syndrome and FHIC have been described and observed in patients, the uveitis typically presents in a unilateral manner. To the best of our knowledge, a case of granulomatous uveitis in a patient with Usher syndrome has not previously been reported. We present a rare finding of bilateral uveitis and what we believe to be the first case of a patient with granulomatous anterior uveitis of unknown etiology in the setting of Usher syndrome.

## Case presentation

A 45-year-old Caucasian woman with RP, previously diagnosed in 2005, presented to our clinic following a referral for suspected Usher syndrome given her history of concomitant high frequency hearing loss. Apart from a mild loss in visual acuity that had been relatively stable for several years, our patient did not have any other significant ocular symptoms, including, most notably, an absence of pain, redness and photophobia. Her medical history was significant for hypertension, for which she was taking a diuretic medication. She was also taking vitamin A supplements. She was not taking any ocular medications and she denied a history of ocular trauma or pathology aside from RP.

Her family history included a 49-year-old sister who was blind with RP and had hearing loss since childhood. Another sister, 47 years old, had a slowly progressive form of tunnel vision with RP (her hearing status was presumed normal). Her two other siblings, a brother and a sister, did not have any vision or hearing impairments.

**Figure 1:**
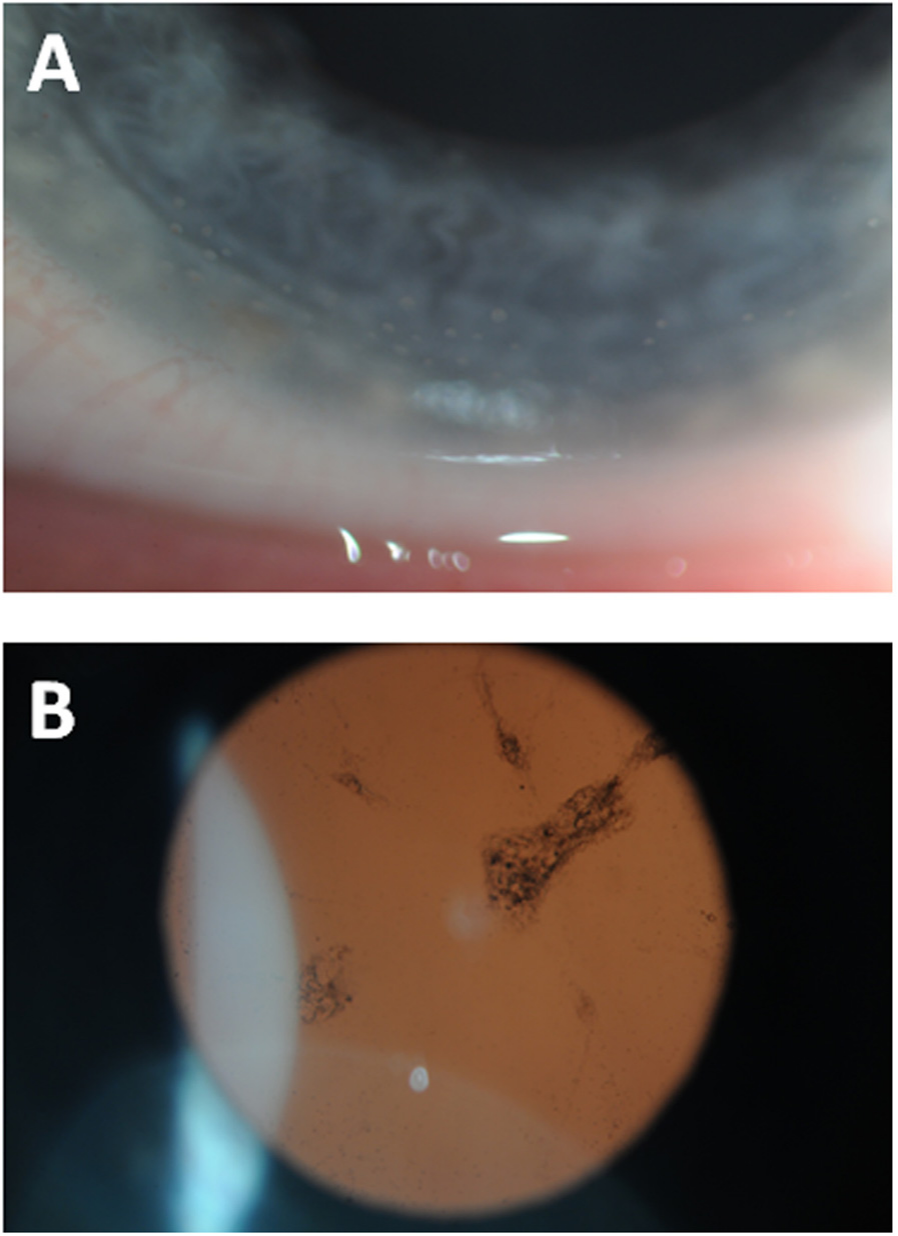
**Slit lamp examination findings in a patient with Usher syndrome. (A)** Anterior segment image with numerous large, greasy-white mutton-fat keratic precipitates seen on the corneal endothelial surface of the left eye. **(B)** Retro-illumination demonstrating a posterior subcapsular cataract.

On examination, our patient had a visual acuity of 20/30+2 in her right eye and 20/30 in her left eye. There was no improvement with pinhole correction. Her intraocular pressures were within normal limits in both eyes. With slit lamp examination, her external examination was normal and her sclera and conjunctiva were not injected. Several small, round KPs were observed on the inferior corneal endothelium in her right eye; however, the anterior chamber was quiet and deep. In the inferior aspect of her left corneal endothelium there were numerous large, greasy-white mutton-fat KPs (Figure 1A). There were 2+ cells based on Standardization of Uveitis Nomenclature classification [12] but no flare in the anterior chamber of her left eye. Her irides were round and flat with no evidence of posterior synechiae or heterochromia. Both eyes had posterior subcapsular cataracts (Figure 1B) and there were occasional pigmented anterior vitreous cells in her left eye. A retinal examination revealed pale optic nerves with arteriolar attenuation and mid-peripheral bone spicule pigment distribution. There were no signs of pars planitis or retinitis with scleral depression. In addition to her known RP, bilateral uveitis with granulomatous uveitis in her left eye was diagnosed based on examination findings.

A macular optical coherence tomography scan demonstrated a preserved macula with no cystoid macular edema. Her previous records indicated an extinguished response with vestigial flicker on electroretinography, suggestive of advanced RP, and moderate bilateral sensorineural hearing loss with severe loss at higher frequencies from an audiology assessment. Our patient had no obvious speech impediments and she did not have cochlear implants. Prior visual field testing had revealed bilateral ring scotomas with central sparing.

A thorough uveitis work-up demonstrated that her angiotensin-converting enzyme, calcium, and liver enzyme levels were within normal limits. She had a slightly elevated erythrocyte sedimentation rate at 25 mm/h (normal range: 0 to 20 mm/h). Tests for human leukocyte antigen B27 and syphilis were negative. A chest X-ray showed no definite hilar or mediastinal lymphadenopathy and no signs of tuberculosis. Findings from an X-ray of her sacroiliac joints were normal.

Based on these results, our patient was diagnosed with type 2 Usher syndrome with an associated granulomatous uveitis of undetermined etiology. Given the absence of symptoms and apparent chronicity of presentation, treatment was not indicated at the time of her presentation. She was encouraged to attend follow-up appointments to monitor the progression of her disease and any possible complications of the uveitis. Formal genetic testing was discussed, but access to clinical testing is generally problematic and research testing has yet to be initiated.

## Discussion

Usher syndrome is a genetically heterogeneous condition that represents the most common cause of inherited deafness and blindness. Individuals typically present with marked sensorineural hearing loss and vision impairment secondary to retinitis pigmentosa. The differentiation of the forms of Usher syndrome based solely on symptoms may be problematic and not lead to a specific diagnosis. Increasingly molecular genetic analysis is called upon, when available on a clinical basis, to guide the investigation of these cases. To date, fourteen gene loci have been implicated in Usher syndrome with ten of these genes being identified [13]. Inherited in an autosomal recessive pattern, mutations in these genes can lead to functional impairment with significant quality of life implications, making this an important condition to recognize and accurately diagnose.

Several studies have demonstrated an association between RP, the main ocular phenotype in Usher syndrome, and FHIC [5-9]. The presentation of anterior uveitis in FHIC is typically unilateral and chronic, with patients often lacking any symptoms. A characteristic slit-lamp finding involves stellate KPs on the corneal endothelium, providing further evidence for this non-granulomatous form of uveitis. While the majority of studies that observed FHIC in patients with RP described unilateral KPs as expected, Sandinha *et al.* reported a case of bilateral stellate KPs [8].

More recently, studies have demonstrated an association between Usher syndrome and FHIC [10,11]. Lichtinger *et al.* performed a retrospective analysis of 58 patients with RP and discovered an even stronger association between FHIC and Usher syndrome. In the two cases presented in that study, both patients had unilateral stellate KPs [10]. In addition, another study demonstrating this association also presented the case of a patient with unilateral stellate KPs [11].

We report the case of a patient with Usher syndrome and bilateral KPs, a rare finding in the literature. Moreover, our patient presented with numerous large, greasy-white mutton-fat KPs on the endothelial surface of her left eye. To the best of our knowledge, this finding has not been previously reported in a patient with Usher syndrome. To date, all but one case of uveitis in Usher syndrome has been attributed to FHIC: Alzuhairy and Alfawaz described a case of non-granulomatous uveitis in a patient with Usher syndrome that did not resemble FHIC [3]. We have, however, been unable to find an observation in the literature of granulomatous uveitis in Usher syndrome. We thus present this rare finding in a recognizable syndrome. It should be mentioned that because this is the first reported instance the possibility of a coincidental association cannot be ruled out.

While the mechanism explaining the association of uveitis and Usher syndrome has not been established, there have been suggestions that an underlying autoimmune reaction may be responsible. Retinal S antigen is present in rod photoreceptors and has been implicated in inflammatory eye conditions including uveitis and retinal disease [14]. Patients with RP have been shown to have B cells that respond and react to retinal S-antigen [15]. It has been suggested that patients with RP, and thus patients with Usher syndrome, may have an underlying tendency for immune reactions that predispose them to developing uveitis. Furthermore, Chowers *et al.* suggest that patients with RP may develop reactions to antigens in the anterior chamber of the eye that can result in uveitis [5]. In their study, they referenced FHIC and suspected other factors involved in the development of uveitis in RP because FHIC typically presents unilaterally. We discovered bilateral KPs in our patient, which adds support to a proposed underlying immune mechanism because uveitis would not be expected to preferentially affect one eye. The granulomatous reaction seen in our patient does, however, suggest that other factors may exist and contribute to uveitis in Usher syndrome.

## Conclusion

We have reviewed Usher syndrome: a heterogeneous condition that represents the most common inherited cause of combined vision and hearing loss. Deficits typically manifest early on in a patient’s life and the condition can have a significant impact on quality of life. As a result, early detection with an accurate diagnosis is especially important.

While an association between Usher syndrome and non-granulomatous uveitis has been described, our finding of bilateral uveitis is a rare occurrence. Furthermore, to the best of our knowledge, we are the first to report granulomatous uveitis as a finding in Usher syndrome. Our patient demonstrated numerous mutton-fat KPs on her corneal endothelium that could not be attributed to any known cause. We hope that this illustration of a rather atypical finding in a patient with Usher syndrome may add to the clinical spectrum of ophthalmic conditions that can be seen in association with Usher syndrome.

## Consent

Written informed consent was obtained from the patient for publication of this case report and accompanying images. A copy of the written consent is available for review by the Editor-in-Chief of this journal.

## Abbreviations

FHIC: Fuchs’ heterochromic iridocyclitis
KP: Keratic precipitates
RP: Retinitis pigmentosa
